# Draft genome sequence of two strains of carotenoid-producing yeast *Rhodotorula* sp., isolated from a mangrove leaf in Okinawa, Japan

**DOI:** 10.1128/mra.00087-26

**Published:** 2026-06-22

**Authors:** Anh Thi Nhat Tran, Toshikazu Suenaga, Takehiko Gotoh, Wataru Nishijima, Satoshi Nakai

**Affiliations:** 1 Department of Chemical Engineering, Hiroshima University 12803, Higashi-Hiroshima, Hiroshima, Japan; 2 Environmental Research and Management Center, Hiroshima University 68272, Higashi-Hiroshima, Hiroshima, Japan; University of California Riverside, Riverside, California, USA

**Keywords:** *Rhodororula* sp., yeasts, genomics

## Abstract

Basidiomycetous red yeast *Rhodotorula* sp. is prevalent and abundant in the open ocean. This study reports the draft genomes of the *Rhodotorula* sp. strains HGY1 and HGY2. The strains are capable of producing carotenoids, such as β-carotene, and are regarded as potential hosts for biorefinery.

## ANNOUNCEMENT

*Rhodotorula* is a genus of red yeast that belongs to the phylum Basidiomycota (family Sporidiobolaceae) and inhabits diverse environments and clinical settings (1). Strains of this genus have been studied because of their ability to accumulate antioxidant carotenoids, including β-carotene and astaxanthin (2, 3), as well as and fatty acids and enzymes (4, 5). Notably, a number of *Rhodotorula* species possess psychrophilic and extremophilic characteristics that allow them to survive at low temperatures and low pH levels (6, 7). Consequently, sequencing the genomes of novel *Rhodotorula* strains enables comparative analyses among yeasts and provides insights into their genetic basis.

*Rhodotorula* sp. strains HGY1 and HGY2 were isolated from mangrove leaf in Okinawa (26°21′49.5″ N 127°44′47.2″ E; 2018), Japan, using the same procedure as *Aurantiochytrium* sp. strains described in a previous study (8). We confirmed the production of carotenoids such as β-carotene in these strains by HPLC analysis (0.03 and 0.06 mg/g biomass, respectively). Leaf samples were aseptically placed on antibiotic-supplemented ATCC 2673 agar and incubated at 25°C in the dark. Emerging colonies were then transferred to ATCC 790 By+ liquid medium and cultivated with shaking for strain isolation (9). Purified strains were obtained by repeated single-colony isolation after initial plating on ATCC 2673 agar. Colonies were serially diluted and re-plated until axenic cultures were established (9). Cells were lysed and the genome was extracted using the ISOIL Beads Beating DNA Extraction Kit (2nd edition, Nippon Gene, Tokyo, Japan) following the manufacturer’s protocols. The DNA was enzymatically sheared into short fragments. The library was prepared from the DNA fragments using the Rapid Plus DNA Lib Prep Kit for Illumina v2 (ABclonal, Hubei-China), MGIEasy Circularization Kit, and MGIEasy Universal Library Conversion Kit (MGI_Tech., Shenzhen, China) for end repair, A-tailing, adapter ligation, and circularization. Sequencing data were collected by DNBSEQ T7 platform with 150 × 150 paired-end sequencing mode by the sequencing service (Novogene, Beijing, China). Poor-quality reads (<Q30) and sequencing adapters were removed with fastp (v.0.23.2) (10) and assembled by MEGAHIT (v.2.1.9) using default parameters (11). The repeat sequences were masked using Red (v.2.0) (12). Genome completeness was evaluated using BUSCO (v6.0.0) (13). Information on the sequencing data and genome is summarized in Table 1.

**TABLE 1:** General genomic characteristics of *Rhodotorula* sp. strain HGY1, strain HGY2

Strain name	*Rhodotorula* sp. strain HGY1	*Rhodotorula* sp. strain HGY2
The closest species based on BLAST search of 18S rRNA (percent identity [%])	*Rhodotorula diobovata* strain JCM 3787 (99.55)	*Rhodotorula diobovata* strain JCM 3787 (99.55)
Genome size (Mbp)	21.2	21.4
GC content (%)	64.24	64.28
Number of contigs	441	339
*N*_50_ of assembly (kbp)	241	243
Output number of reads (×10^7^ reads)	1.96	1.33
Output bases (Gbp)	2.8	2.0
Coverage (×)	132	93
BUSCO score (%)	96.1	94.6
Acc. num. of genome assembly	BAAJPB010000000	BAAJPC010000000
Acc. num. of BioSample	SAMD0179279	SAMD01797280
Acc. num. of SRA	DRR899617	DRR899618

**Fig 1:**
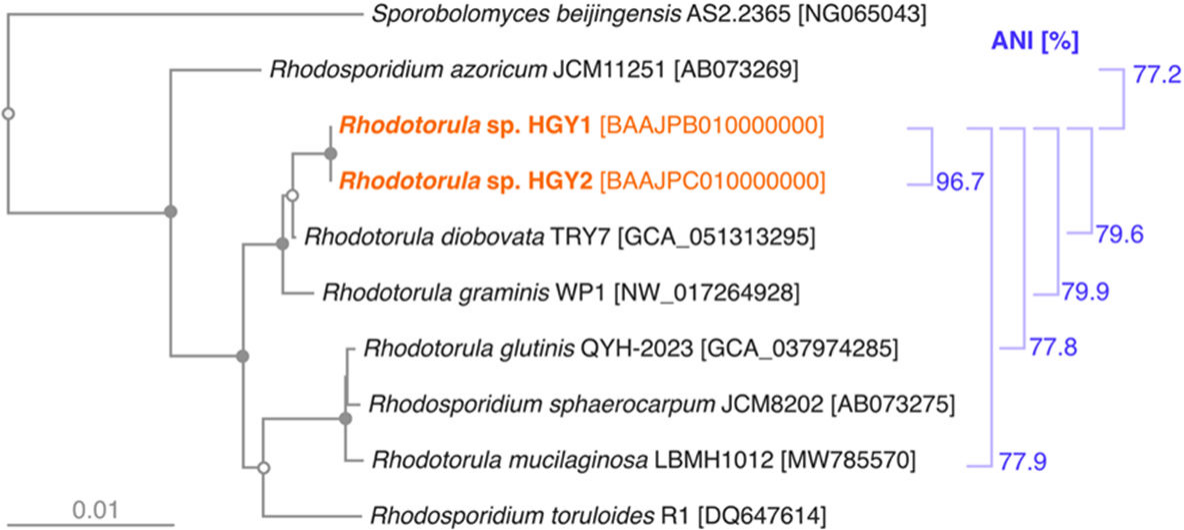
Phylogenetic tree based on full-length 18S rRNA gene sequences. The strains used in this investigation (*Rhodotorula* sp. HGY1 and HGY2) are shown in bold. Bootstrap values were obtained using 100 replicates and are shown as >75% (closed circles) and 50–75% (open circles) at each node. The sequences were aligned using MAFFT, and the phylogenetic tree was inferred using the neighbor-joining method. The tree was visualized with Archaeopteryx.js v.2.0.0 and then improved graphically with Affinity (v.3.0.2). The ANI values for each comparison between strain HGY1 and the other strains are shown in blue.

The total length was estimated to be 21.2–21.4 Mbp, close to that of related species reported for *R. diobovata* strain 08-225 (21.1 Mbp) (14) and *R. glutinis* strain ZHK (22.3 Mbp) (15). Figure 1 shows the phylogenetic tree of the isolated strains based on full-length 18S rRNA sequences, aligned using MAFFT_v.7 (16). These two strains (HGY1 and HGY2) were closely related to *Rhodotorula diobovata* strain JCM 3787, showing an identity score of 99.55% based on a BLAST search (17) of the 18S rRNA sequence. The ANI values calculated using FastANI (18) are also shown in Fig. 1, indicating that these strains belong to the genus *Rhodotorula*. The genomes reported in this study are promising to contribute to the elucidation of the metabolism of bio-products in industrial applications of *Rhodotorula* sp.

## Data Availability

The sequencing data were deposited in DDBJ under BioProject ID PRJDB40089, and the accession numbers of the genomes of strains HGY1 and HGY2 are BAAJPB010000001–BAAJPB010000441 and BAAJPC010000001–BAAJPC010000339, respectively.
